# A Chromatin-Focused siRNA Screen for Regulators of p53-Dependent Transcription

**DOI:** 10.1534/g3.116.031534

**Published:** 2016-06-20

**Authors:** Morgan A. Sammons, Jiajun Zhu, Shelley L. Berger

**Affiliations:** Epigenetics Program, Department of Cell and Developmental Biology, Perelman School of Medicine, University of Pennsylvania, Philadelphia, Pennsylvania 19104

**Keywords:** chromatin, p53, siRNA screen, epigenetics, *H. sapiens*

## Abstract

The protein product of the *Homo sapiens TP53* gene is a transcription factor (p53) that regulates the expression of genes critical for the response to DNA damage and tumor suppression, including genes involved in cell cycle arrest, apoptosis, DNA repair, metabolism, and a number of other tumorigenesis-related pathways. Differential transcriptional regulation of these genes is believed to alter the balance between two p53-dependent cell fates: cell cycle arrest or apoptosis. A number of previously identified p53 cofactors covalently modify and alter the function of both the p53 protein and histone proteins. Both gain- and loss-of-function mutations in chromatin modifiers have been strongly implicated in cancer development; thus, we sought to identify novel chromatin regulatory proteins that affect p53-dependent transcription and the balance between the expression of pro-cell cycle arrest and proapoptotic genes. We utilized an siRNA library designed against predicted chromatin regulatory proteins, and identified known and novel chromatin-related factors that affect both global p53-dependent transcription and gene-specific regulators of p53 transcriptional activation. The results from this screen will serve as a comprehensive resource for those interested in further characterizing chromatin and epigenetic factors that regulate p53 transcription.

The p53 protein (encoded by the *TP53* gene in *Homo sapiens*) is a sequence-specific transcription factor and a master tumor suppressor. Inactivating *TP53* mutations are observed in over 50% of human cancers, and the loss of p53 activity leads to genome instability and metabolic dysfunction, ultimately promoting tumor formation (Lawrence *et al.* 2014). p53 is activated in response to a number of diverse cellular stress signals, including DNA damage, oncogene activation, loss of normal metabolic homeostasis, and telomere attrition, and mediates the expression of cell- and organismal-protective genes involved in processes such as DNA repair, cell cycle arrest, and apoptosis (Kruiswijk *et al.* 2015).

p53 is negatively regulated through direct interaction and ubiquitination by the E3-ubiquitin ligase MDM2, which mediates proteosomal degradation of p53 during nonstress conditions (Momand *et al.* 1992; Oliner *et al.* 1992; Wu *et al.* 1993). Upon stress, the p53:MDM2 complex is disrupted by kinases like ATM, which directly phosphorylate p53 in the MDM2 interaction domain, leading to stabilization and upregulation of the p53 protein level (Siliciano *et al.* 1997; Shieh *et al.* 1997). The transcriptional activity of p53 can be modulated by other cofactors, including a number of other enzymes that directly catalyze posttranslational modifications such as acetylation and methylation on various amino acids (Berger 2010; Dai and Gu 2010). Such modifications may act to fine-tune the p53 response, either through altering the DNA binding specificity of p53 (Luo *et al.* 2004) or through recruitment of p53 interacting proteins, like 53BP1 (Tong *et al.* 2015a,b; Barlev *et al.* 2001).

Many p53 modifying enzymes, such as *PCAF*/GCN5, *SETD8*/PR-Set7, and *SMYD2*, also play critical roles in transcriptional regulation through direct modification of histone proteins (Liu *et al.* 1999; Huang *et al.* 2006; Shi *et al.* 2007). A number of these chromatin regulatory factors and pathways have also been implicated in the maintenance of normal homeostasis, with mutated or hyperactive chromatin modifying enzymes and pathways contributing to disease development, including many cancers (Bannister and Kouzarides 2011; Kouzarides 2007). Given the importance of chromatin regulatory factors in regulating normal and disease-associated transcriptional responses, many small molecule inhibitors are being developed as putative therapeutics for various diseases (Dawson and Kouzarides 2012; Dawson *et al.* 2012).

Importantly, in response to cellular damage, p53 coordinates the transcription of factors involved in potential oncogenic transformation. The ultimate transcriptional output of p53 leads to a modulation of cell fate; a p53-activated cell can undergo transient cell-cycle arrest until the damage/stress is alleviated, permanent cell cycle arrest (also known as senescence) (Rufini *et al.* 2013), or cell death via apoptosis (Zilfou and Lowe 2009). The molecular mechanisms that modulate these alternative transcriptional programs have been an area of intense investigation (Andrysik *et al.* 2013). Of particular interest are chromatin and protein modifications that may underlie the differential transcription. For example, the lysine acetyltransferase KAT5/TIP60 influences transcription of the proapoptotic p53 target gene BBC3/puma without affecting the transcription of prosurvival p53 targets like *CDKN1A*/p21. *KAT5*/TIP60 also directly acetylates p53 at lysine 120, suggesting that this modification affects p53 target gene discrimination (Sykes *et al.* 2006; Tang *et al.* 2006). These and other results strongly implicate p53 modification and chromatin pathways in the regulation of key pathways underlying the fate of damaged cells; however, the specific mechanisms that result in this fate choice remain elusive.

As discussed above, a number of enzymes capable of directly modifying the p53 protein have been implicated in general regulation of p53-dependent transcription. In addition, we and others observed that p53 binds directly to DNA in a varied chromatin and *cis*-regulatory element context (Lidor Nili *et al.* 2010; Sammons *et al.* 2015; Su *et al.* 2015), suggesting that chromatin structure and modifications might directly influence p53 activity. We hypothesized that chromatin and epigenetic regulatory mechanisms might modulate p53-dependent transcription and tumor suppression. Thus, we designed the siRNA screen to: 1) identify chromatin and epigenetic regulatory proteins that modulate p53-dependent transcription; and 2) identify new *trans*-acting factors that could influence the ability of p53 to enact a prosurvival (*CDKN1A*/p21) or proapoptotic (*BBC3*/puma) transcriptional program. The results uncovered from this screen provide a strong basis for future studies focused on characterizing key mechanisms underlying p53-mediated cell fate regulation.

## Materials and Methods

### siRNA screen design

The human osteosarcoma cell line U2OS (HTB-96, ATCC) was grown in McCoy’s 5A medium (supplemented with 10% fetal bovine serum and penicillin/streptomycin) at 37° in a standard CO_2_ incubator. A custom Thermo SmartPool siRNA library targeting chromatin regulatory genes (Supplemental Material, Table S1) was arrayed on a 384-well plate and resuspended in 1 × siRNA Buffer (Dharmacon) at 1 μM. Chromatin regulator targets were manually curated based on a previously published list of putative chromatin regulatory factors (Zuber *et al.* 2011). Human genes containing domains with previously characterized chromatin regulatory activity (*i.e.*, SET, PHD, Bromo domains, Chromo domains, etc.) were identified using PFAM (EMBL-EBI). All human genes containing these domains were included, even if previously not implicated directly in chromatin regulatory function. Kinases and phosphatases with previously demonstrated direct chromatin regulatory activity were included, and those without were manually removed from the curation.

siRNA was aliquoted into single use 96-well plates with internal control siRNA against TP53, MDM2, and a nonspecific targeting siRNA. Each siRNA was delivered to 11,000 cells via reverse transfection using RNAiMax (Life Technologies) in a 96-well plate to a final concentration of 10 μM, media was changed after 24 hr, and cells were incubated for an additional 48 hr before addition of either DMSO or 100 μM (final) etoposide (Sigma-Aldrich) for 8 hr. PolyA+ RNA was isolated using mRNA Catcher (Life Technologies) and cDNA synthesis was performed on-plate using random hexamer priming. Triplicate qPCR reactions containing cDNA, PowerSybr qPCR Mastermix (Life Technologies), and gene-specific primer pairs were loaded into 384-well plates using an Eppendorf EpMotion 5070, and target gene expression was measured using the relative standard curve method on an ABI 7900HT PCR instrument (Applied Biosystems).

### Data analysis and scoring of hits

Target-specific gene expression values for each siRNA knockdown were normalized to mRNA expression of *LMNA*/lamin A/C. Standard scores (*z*-scores) were calculated using the equation z=(X−μ)/θ, where *X* = gene expression value for an individual target, *μ* = mean gene expression value across all siRNA experiments, and *θ* = standard deviation of gene expression values across all siRNA experiments. Values with a *z*-score representing ± 2 deviations from the standard score were called as hits. Clustering was performed using a 5 × 5 self-organizing map (SOM) with 100 training iterations and implemented using the *kohonen* package in R (Wehrens and Buydens 2007). Table S1 contains a complete list of gene targets, normalized expression values, and SOM clusters.

### Rescreening of a subpool of siRNA and analysis of potential false positives

A random selection of 81 siRNAs were rescreened for their ability to modulate *CDKN1A*/p21 and *BBC3*/puma using the same methodology as above with the following changes. First, we screened only using the DMSO/basal condition. Second, we reduced the cut-off for calling a hit in the secondary screen to 1 standard deviation from the mean. Hits present in both the primary and secondary screen are marked with an ampersand in Table 2, Table 3, and Table 4. Putative false positives were then called if they were present but not called as hits in the rescreening experiment. Rescreen expression values can be found in Table S1 under the Secondary Screen tab. Additionally, putative false positives were called if the corresponding expression value for that gene had a value of 0 (not expressed) from a published RNA-seq gene expression analysis (Klijn *et al.* 2015). Expression values for each gene in the siRNA screen can be found in Table S1. Putative false positives identified using these two methods are now marked with asterisks in Table 2, Table 3, Table 4, and Table 5.

### Data availability

Table S1 contains all normalized gene expression data across all screening conditions. Screen data are also available at the GenomeRNAi repository under accession number GR00389-S.

## Results and Discussion

### Chromatin-focused siRNA screen performance

Based on previous observations of overlap between p53 and chromatin regulatory pathways, we used an siRNA-based approach to identify epigenetic or chromatin regulators of p53-dependent transcriptional activity. We designed a targeted “epigenetic bookcase” of siRNA molecules against 589 genes involved in chromatin regulatory mechanisms (Table S1). These genes include known and putative histone modifying enzymes, chromatin remodelers, chaperones, and cofactors. We reasoned that the reduced complexity of this targeted bookcase compared to a full genome library would facilitate the identification of direct p53 regulators, as opposed to chromatin factors that affect pathways far upstream of p53 activity at DNA.

**Figure 1:**
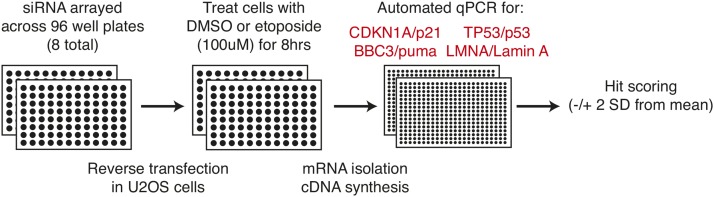
Schematic of steps for reverse transcription-quantitative PCR (RT-qPCR) methodology for screening a chromatin-focused siRNA library. qPCR, quantitative polymerase chain reaction; DMSO, dimethyl sulfoxide; RT-qPCR, reverse transcription-quantitative polymerase chain reaction; SD, standard deviation; siRNA, small interfering RNA.

**Figure 2:**
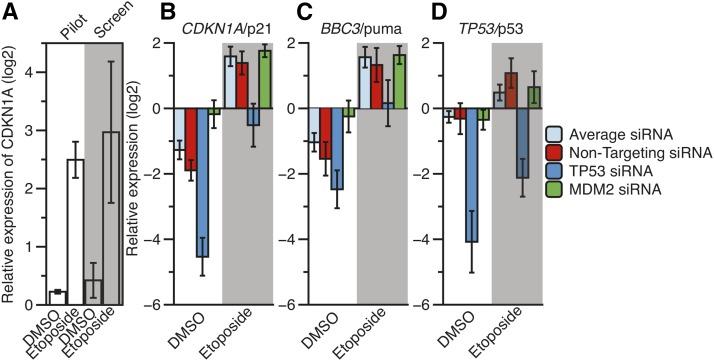
The normalized, relative expression of *CDKN1A*/p21 after DMSO or 8 hr of etoposide treatment (100 μM final) in a pilot RT-qPCR screen and in the experimental siRNA screen (A). Average (B) *CDKN1A*/p21, (C) *BBC3*/puma, and (D) *TP53*/p53 expression values under DMSO and etoposide conditions across each experimental RT-qPCR plate for nontargeting, *TP53*, and *MDM2* siRNA. Results from T-tests of pairwise comparisons can be found in Table 1. DMSO, dimethyl sulfoxide; RT-qPCR, reverse transcription-quantitative polymerase chain reaction; siRNA, small interfering RNA.

We further developed and optimized an automated RT-qPCR-based readout to maximize efficiency and reduce variability. We utilized U2OS osteosarcoma cell lines, which contain wild-type p53 alleles. The workflow measured expression of CDKN1A/p21 in response to p53 activation following 8 hr of treatment with etoposide, a potent DNA damaging agent and p53 activator. Pilot experiments measuring *CDKN1A*/p21 expression in control DMSO or etoposide-treated U2OS cells across 80 biological replicates demonstrated a *z*-score of 0.54, suggesting that this RT-qPCR-based assay would robustly identify putative p53 regulators (Birmingham *et al.* 2009). We screened 589 gene-specific siRNA pools for the ability to modulate the expression of three different p53 target genes (*CDKN1A*/p21, *BBC3*/puma, and *TP53*/p53) following 8 hr of 100 μM etoposide treatment or DMSO control in U2OS cells (Figure 1). We also measured the expression of a control gene (*LMNA*/lamin A). This experimental rationale allowed for the identification of genes that regulate p53-dependent transcription at both the basal level (DMSO) and under activated conditions (etoposide), and would allow for the elimination of those factors whose knockdown resulted in global transcriptional changes in genes unrelated to p53 (lamin A). The targeted siRNA screen measuring *CDKN1A*/p21 transcription mirrored the pilot experiment, although we observed more variation in the siRNA screen, as expected (Figure 2A). Importantly, nontargeting siRNA and control siRNA treatments targeting *TP53* or *MDM2* produced expected results, indicating that the siRNAs approach was feasible and successful. Nontargeting siRNA displayed comparable expression values as the average of all experimental targets, as expected (Figure 2, B–D). In contrast, treatment with control *TP53* siRNAs led to severely reduced *CDKN1A*/p21, *BBC3*/puma, and *TP53*/p53 expression (Figure 2, B–D). *MDM2* siRNA controls resulted in increased basal CDKN1A and BBC3 expression, but not TP53, as expected of the role of *MDM2* in posttranscriptional regulation of p53 protein activity (Figure 2B). Results of statistical tests for pairwise comparison of control siRNA experiments can be found in Table 1.

**Table 1:** Results of T-tests for screen-average siRNA related to Figure 2

	CDKN1A DMSO	CDKN1A Etoposide	BBC3 DMSO	BBC3 Etoposide	TP53 DMSO	TP53 Etoposide
Nontargeting	9.96E-4	0.2407	0.0287	0.2875	0.7663	5.57E-3
TP53	9.54E-10	9.73E-7	1.93E-5	1.51E-4	2.12E-8	1.20E-8
MDM2	3.12E-5	0.1946	1.43E-3	0.6630	0.4927	0.4037

DMSO, dimethyl sulfoxide.

**Figure 3:**
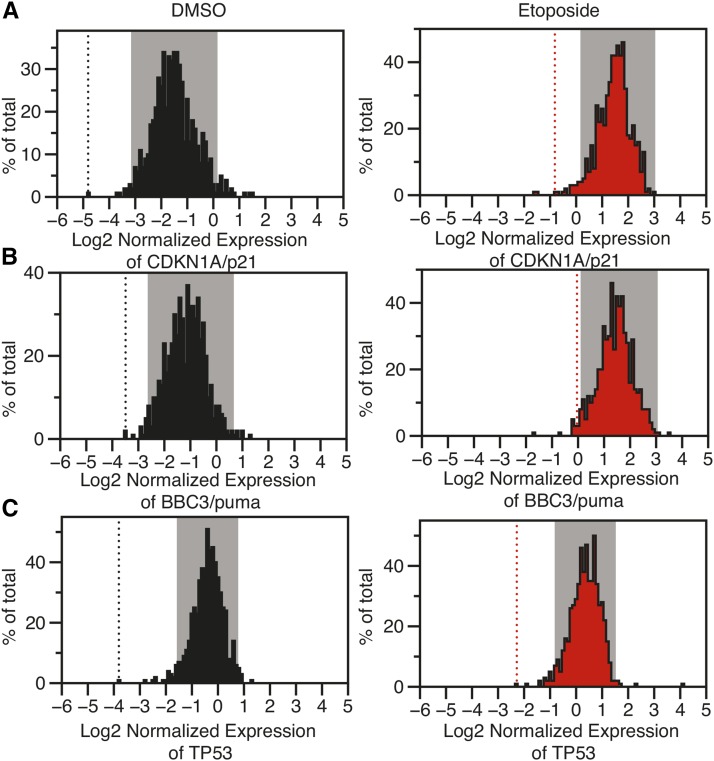
The distribution of relative RNA expression for (A) *CDKN1A*/p21, (B) *BBC3*/puma, and (C) *TP53*/p53. Gray windows represent data with *z*-scores between –2 and 2. Dotted lines represent the expression value for *TP53* knockdown siRNA used as an internal control within the screen. siRNA, small interfering RNA.

Normalized gene expression values for each of the target mRNA molecules were normally distributed (Figure 3). Therefore, we used a *z*-score-based cut-off to call hits as siRNA treatments that altered expression ± 2 standard deviations from the mean of all expression values for that target gene (Birmingham *et al.* 2009). Gray boxes in Figure 3 depict gene expression values that fall within 2 standard deviations from the mean, and are thus not called as hits. As an important control, we note that *TP53* siRNA, included in our bookcase (Table S1), scored as a hit for all six conditions tested using these criteria, and are presented as dashed lines in Figure 3.

### Positive regulators of CDKN1A/p21 and BBC3/puma

We identified 10 genes that behave as putative positive regulators of both *CDKN1A*/p21 and *BBC3*/puma transcription (Table 2). Based on the scoring strategy described above, these genes have *z*-scores < −2 for both *CDKN1A*/p21 and *BBC3*/puma, either in the basal (DMSO-treated) condition or after etoposide treatment. As expected, knockdown of TP53 itself led to the most dramatic decrease in basal CDKN1A and BBC3 expression of all genes tested (Figure 3, A and B, DMSO). PLK1 knockdown resulted in a significant reduction in CDKN1A, BBC3, and TP53 mRNA, suggesting potentially indirect effects on p53 target gene transcription through reduced p53 protein expression. siRNA-mediated knockdown of SETD3 and NCAPG reduced CDKN1A and BBC3 mRNA expression under basal conditions (DMSO), but not upon etoposide treatment. Interestingly, TOP1 (topoisomerase 1) scored as a positive regulator of p53 by reducing p53 target gene transcription after treatment with etoposide. This is of particular interest, since etoposide is a potent topoisomerase II poison that acts to inhibit repair of TopoII-induced dsDNA breaks, which, in turn, is a potent activator of p53-dependent signaling (Fortune and Osheroff 2000; Pommier *et al.* 2010). Both TOP1 and TOP2 have been implicated directly in positive gene regulation through their ability to relax DNA coiling (King *et al.* 2013; Madabhushi *et al.* 2015). Therefore, alternative p53-activating stimuli like the MDM2 inhibitor nutlin 3A (Vassilev *et al.* 2004) may prove to be useful in further investigating the roles for TOP1 and TOP2 in regulating p53-dependent transcription.

**Table 2:** Regulators of *CDKN1A*/p21 and *BBC3*/puma

Downregulated by siRNA		Upregulated by siRNA	
(Positive Regulator)		(Negative Regulator)	
DMSO	Etoposide	DMSO	Etoposide
TP53	PLK1	CTCF^&^	JHDM1D
SETD3	TP53	MLL2	
NCAPG	PRMT6	YEATS4	
	MBD3L2*	TRERF1	
	TOP1	EP300^&^	
	SETDB1	SRCAP^&^	
	PHC1	USP27X	
		HIRIP3^&^	
		KIAA1267^&^	

Genes are listed in order of strongest to weakest phenotypic effect. Full phenotypic values can be found in Table S1. * and ^&^ denote putative false positives and secondary screen hits, respectively. Information about identification of putative false positives and secondary screen hits can be found in *Materials and Methods*. siRNA, small interfering RNA; DMSO, dimethyl sulfoxide.

### Negative regulators of CDKN1A/p21 and BBC3/puma

We identified 10 genes that are predicted to act as negative regulators of both *CDKN1A*/p21 and *BBC3*/puma expression, with mRNA expression *z*-scores > 2 for both genes (Table 2). Nine of these 10 genes increased CDKN1A and BBC3 mRNA expression under basal conditions; only *JHDM1D*/KDM7A further increased activation of *CDKN1A*/p21 and *BBC3*/puma after etoposide treatment. JHDM1D catalyzes the removal of dimethylation from lysine 9 of histone H3 (H3K9me2) (Horton *et al.* 2010), a canonically repressive histone modification catalyzed by SETDB1 (Schultz *et al.* 2002), which was identified above as a positive regulator of p53 transcription. SETDB1 and JHDM1D regulate transcription through respective deposition and removal of heterochromatin-associated histone modifications, an activity that is predicted to have an opposite effect on p53-dependent transcription than what is observed in this screen. Further investigation is required to determine whether SETDB1 and JHDM1D control p53 through transcription-associated histone modifications or through direct interaction/modification of p53.

Knockdown of the boundary element and chromatin looping factor CTCF has been previously shown to derepress *BBC3*/puma transcription in the basal state (Gomes and Espinosa 2010). We observed that siRNA-mediated knockdown of CTCF led to upregulation of *BBC3*/puma, as well as derepression of CDKN1A/p21, further suggesting that chromatin looping may function in the regulation of p53 targets (Merkenschlager and Odom 2013; Kim *et al.* 2015). The SRCAP complex, which catalyzes exchange of H2A.Z for H2A in nucleosomes, contains both the catalytic protein SRCAP and *YEATS4*/GAS41, a previously identified repressor of p53 activity (Park and Roeder 2006; Wong *et al.* 2007; Pikor *et al.* 2013). In our screen, knockdown of either *SRCAP* or *YEATS4*/GAS41 led to increased mRNA expression of p53 target genes. SRCAP represses ΔNp63 target genes through its H2A.Z deposition activity (Gallant-Behm *et al.* 2012) and appears to behave similarly with p53 transcriptional targets in our screen. This suggests a potential common repressive mechanism for p53 family transcription factors through SRCAP complex-mediated H2A.Z deposition.

### Specific regulators of CDKN1A/p21, BBC3/puma, or TP53/p53

We next examined genes that behaved as gene-specific regulators of *CDKN1A*/p21, *BBC3*/puma, or *TP53*/p53 (Table 3, Table 4, and Table 5). Strikingly, there were many siRNA targets that were specific for either *CDKN1A*/p21 or *BBC3*/puma. For example, *KAT5*/TIP60 is a lysine acetyltransferase that catalyzes the acetylation of histone H4 lysine 16 (H4K16ac) and is critical for DNA repair (Tang *et al.* 2013). Beyond the canonical role in histone acetylation and DNA damage, *KAT5*/TIP60 directly interacts with and acetylates p53 at lysine 120. This activity modulates the ability of p53 to activate *BBC3*/puma, but not *CDKN1A*/p21 (Sykes *et al.* 2006; Tang *et al.* 2006). Consistent with these data, KAT5 knockdown in our screen specifically inhibited etoposide-induced *BBC3*/puma expression but not expression of CDKN1A/p21. Similarly, knockdown of *SNW1*/SKIP1 affected only *CDKN1A*/p21 mRNA expression, consistent with reports of SNW1 binding to the CDKN1A gene to regulate cotranscriptional splicing (Chen *et al.* 2011).

**Table 3:** Specific regulators of CDKN1A/p21

Downregulated by siRNA		Upregulated by siRNA	
(Positive Regulator)		(Negative Regulator)	
DMSO	Etoposide	DMSO	Etoposide
TBL1XR1	PCGF2^&^	EIF2S3	CHMP4A
CBX7^&^	PRMT2	SNAPC4	FKBP1A
RCOR2^&^	SMC2	CHEK1^&^	
MBD6*	CBX7	HMG20B^&^	
PCGF2^&^	TNP1*	CDY1B*	
SMC1B	NSD1	SETD8	
FBXW7	PCMT1	KDM4A	
	TBL1XR1	YWHAE	
	SMC1A	CDC5L^&^	
	SMC1B	SLBP^&^	
	SATB1	FOXA2	
	HMGA2		
	SNW1		
	TCF7L2		
	MECP2		

Genes are listed in order of strongest to weakest phenotypic effect. Full phenotypic values can be found in Table S1. * and ^&^ denote putative false positives and secondary screen hits, respectively. Information about identification of putative false positives and secondary screen hits can be found in *Materials and Methods*. siRNA, small interfering RNA; DMSO, dimethyl sulfoxide.

**Table 4:** Specific regulators of BBC3/puma

Downregulated by siRNA		Upregulated by siRNA	
(Positive Regulator)		(Negative Regulator)	
DMSO	Etoposide	DMSO	Etoposide
MCM2	SUDS3	DLX2*	BAZ1B
SFMBT1	SETD8	HMGN2	DACH1
MBD3L1*	TADA2A	ZNF24^&^	MBD2
SETD1B	KAT5	NUP62^&^	DLX2*
CEBPB	TCF7L1	RARA	DMPK
PRMT3	NAP1LF		WNT5A
FGF19*	SMARCA5		YWHAB
NDEL1	PAM		SOX12
PRMT5	MCM10		
	SMC4		

Genes are listed in order of strongest to weakest phenotypic effect. Full phenotypic values can be found in Table S1. * and ^&^ denote putative false positives and secondary screen hits, respectively. Information about identification of putative false positives and secondary screen hits can be found in *Materials and Methods*. siRNA, small interfering RNA; DMSO, dimethyl sulfoxide.

**Table 5:** Specific regulators of *TP53*/p53

Downregulated by siRNA		Upregulated by siRNA	
(Positive Regulator)		(Negative Regulator)	
DMSO	Etoposide	DMSO	Etoposide
LMNB1	SMYD3	ERCC6	UBE2I
ERCC6	PRMT8	RNF20	DIDO1
HMGN1	JUN	PHF5A	CDHD9
FKBP2	PRDM1	MLL5	DFFB
HDAC9	NFKB1	SMARCD2	
SIN3A	KAT2A	HMGN4	
ATM	NCOA3	HMGN1	
CREB1		HMGN1	
KDM5D		CXXC1	
FOXC2		SIN3A	
HDAC6		HDAC1	
		SFMBT2	
		ESR2	

Genes are listed in order of strongest to weakest phenotypic effect. Full phenotypic values can be found in Table S1. * and ^&^ denote putative false positives and secondary screen hits, respectively. Information about identification of putative false positives and secondary screen hits can be found in *Materials and Methods*. siRNA, small interfering RNA; DMSO, dimethyl sulfoxide.

Treatment of U2OS cells with siRNA directed against the product of the *ERCC6* gene, a protein commonly known as CSB, led to a specific reduction of TP53 expression in both the DMSO and etoposide conditions without affecting downstream p53 targets (Table 5). *ERCC6*/CSB mutations lead to Cockayne Syndrome, which is characterized by nervous system and DNA damage, and premature aging phenotypes (Mallery *et al.* 1998). Reduction in TP53 expression after treatment with siRNA targeting *ERCC6*/CSB is consistent with observations that CSB partially regulates the DNA damage response (Proietti-De-Santis *et al.* 2006) and directly interacts with the p53 protein *in vivo* (Latini *et al.* 2011; Lake *et al.* 2011) We observed that siRNA treatment against *SMYD2*/SMYD2 led to an upregulation of TP53 mRNA after DMSO treatment (Table 5). SMYD2 catalyzes the methylation of the p53 protein at lysine 370, which represses p53-dependent transcription (Huang *et al.* 2006). Our data suggests that a number of known and unknown chromatin regulatory proteins, such as ERCC6/CSB and SMYD2, may influence TP53/p53 mRNA levels without a concomitant regulation of downstream p53 target genes.

CBX7 and PCGF2 are both members of the polycomb group family complex PRC1, and their reduction by siRNA treatment strongly diminished *CDKN1A*/p21 mRNA expression without affecting *BBC3*/puma (Table 3). Canonically, polycomb group complexes regulate facultative heterochromatin and mediate gene repression (Di Croce and Helin 2013), and CBX7 is a tumor suppressor in the hematopoeitic lineage (Fortune and Osheroff 2000; Klauke *et al.* 2013). Our results suggest a novel role for PRC1 complexes as potential direct coactivators for p53-mediated transcription of CDKN1A; however, we cannot rule out an indirect role of PCGF2 and CBX7 knockdown leading to derepression of a *CDKN1A*/p21 repressor. Further investigation is required to fully characterize the role of PRC1-mediated p53-dependent transcription regulation.

### Self-organizing map clustering to identify genes with similar p53-dependent transcriptional profiles

**Figure 4:**
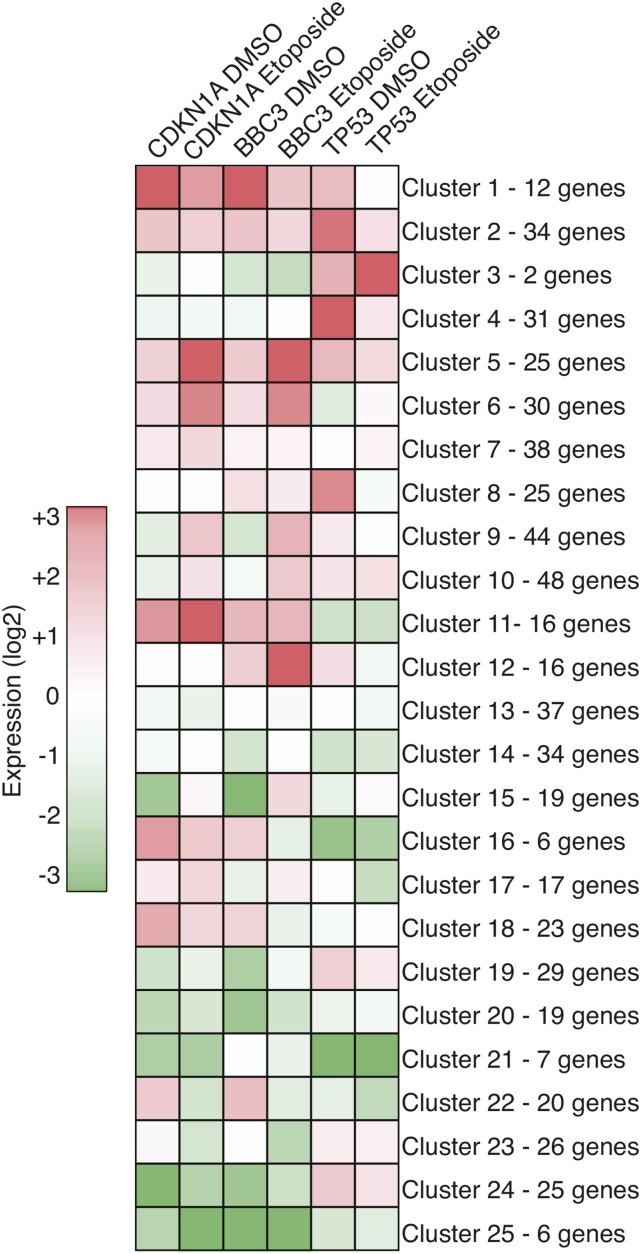
A self-organizing map (SOM) analysis was used to create 25 clusters that behave similarly across the six gene expression values. All clustering data can be found in Table S1. DMSO, dimethyl sulfoxide.

Following review of the screen results, we reasoned that using a stringent *z*-score cut-off to call screen hits might preclude identification of potential p53 regulators that behave similarly to known regulators, or other novel hits within the screen, but fall outside of the *z*-score cut-off. Therefore, we used normalized expression values of *CDKN1A*/p21, *BBC3*/puma, and *TP53*/p53 under both DMSO and etoposide treatment conditions as the input for self-organizing map (SOM) analysis (Kohonen 1987; Wehrens and Buydens 2007) to define groups of genes that similarly regulate p53 target genes (Figure 4 and Table S1).

TP53 siRNA was grouped into Cluster 21 with ATM, ERCC6, HMGA2, LMNB1, PRMT2, and SIN3A siRNA. This cluster is characterized by the lowest levels of TP53 expression across all tested siRNA and reduced CDKN1A/p21 and BBC3/puma levels. Thus, these genes may function as positive regulators of TP53 transcription and act upstream of direct regulation of CDKN1A/p21 or BBC3/puma. ATM was identified as a hit using our initial hit criteria and clusters here with TP53. ERCC6, a chromatin remodeling protein also known as CSB, has previously been implicated in positive regulation of p53 transcriptional activity (Lake *et al.* 2011). In contrast, Cluster 25 contained six genes (MBD3L2, PLK1, PRMT6, SETD1B, SMC1A, and TOP1) that displayed reduced CDKN1A/p21 and BBC3/puma expression and also likely act as positive regulators of TP53-dependent transcription, but without reduced TP53 expression.

Cluster 1 contained 12 genes that are characterized by a dramatic increase in basal expression of CDKN1A and BBC3, yet with only moderate effects on TP53 expression. CTCF and the SRCAP chromatin remodeling complex members (YEATS4 and SRCAP; discussed above) are grouped into this cluster, along with eight genes that were identified as hits using the *z*-score-based cut-off. Interestingly, SOM analysis places ATG7 into Cluster 1, despite failing to be called a hit using the strict *z*-score cut-off criteria. ATG7 was previously described as a direct p53 binding protein, and a loss of ATG7 led to increased BBC3/puma expression, similar to the results of our primary screen (Lee *et al.* 2012).

In contrast, Cluster 5 is specific for genes that negatively regulate etoposide-induced p53 transcription of CDKN1A and BBC3, as siRNA-mediated knockdown of these genes leads to increased expression of CDKN1A and BBC3. Clusters 11 and 12 can be characterized as negative regulators of etoposide-induced CDKN1A or BBC3 transcription, respectively. Overall, these SOM analysis-derived clusters can serve as an alternative reference for selecting putative regulators of gene-specific p53 transcription for further study.

We performed limited validation by rescreening 81 random siRNA targets for *CDKN1A*/p21 and *BBC3*/puma expression after DMSO treatment. We reasoned that random selection of siRNA targets would not skew the distribution of expression values and would allow us to use *z*-score-based hit selection, similar to our original scoring system. A total of 19 putative *CDKN1A*/p21 or *BBC3*/puma regulators were present in the rescreen. Overall, 17 out of 19 primary hits were called as hits in the secondary screening (marked with ampersands in Table 2, Table 3, and Table 4), with MBD6 and MBD3L1 being called as putative false positives. Consistent with the utility of the approach, MBD3L1 was already listed as a potential false positive due to its low mRNA expression in U2OS cells. DLX2, which was also called as a false positive based on RNA expression, was again scored as a hit in the secondary screen, suggesting strong off-target effects mediated by DLX2-targeting siRNA. Interestingly, although we call DLX2 a false positive based on mRNA expression analysis of U2OS cells, the results of our primary and secondary screen are consistent with the recent discovery that DLX2 interferes with ATM-p53 signaling and functions normally as a negative regulator of p53 activity (Wang *et al.* 2016). It should be noted that, because the same siRNA complexes were used in both the primary and secondary screening approach, orthogonal knockdown approaches and/or siRNA pool deconvolution should be performed in order to truly validate any siRNA target identified in these screens.

In summary, our primary chromatin-focused siRNA screen identified both previously known and putative regulators of p53-dependent transcription. Additional investigation will be required to characterize the specific mechanisms of these enzymes in either directly modulating p53 activity or in gene-specific regulation of the local chromatin environment at CDKN1A, BBC3, or TP53.We note that measurement of mRNA expression as the screen readout does not preclude the possibility that our identified p53 regulatory genes operate at the level of mRNA stability; however, we minimized the influence of other posttranscriptional mechanisms like mRNA translation or protein stability that are possible through measurement of *CDKN1A*/p21 or *BBC3*/puma protein levels. The results of this targeted chromatin siRNA screen will provide a useful foundation and resource for future investigation into the molecular mechanisms regulating p53-dependent transcription and the balance between prosurvival and proapoptotic transcriptional programs.

## Supplementary Material

Supplemental Material
