# Supplementary material for: A Chromatin-Focused siRNA Screen for Regulators of p53-Dependent Transcription
Source: G3 (Bethesda). 2016 Jun 20;6(8):2671–8. doi: 10.1534/g3.116.031534 (PMC4978920; doi:10.1534/g3.116.031534)
Supplement: Supplemental Material [file supp_6_8_2671__index.html]

A Chromatin-Focused siRNA Screen for Regulators of p53-Dependent Transcription — Supplemental Material 

# A Chromatin-Focused siRNA Screen for Regulators of p53-Dependent Transcription

## Supplemental Material for Sammons, Zhu, and Berger, 2016

**Files in this Data Supplement:**

- Table S1 - All normalized gene expression data across all screening conditions. (.xlsx, 197 KB)
